# Eating behaviour and glycaemic control: A cross-sectional study in adolescents with diabetes

**DOI:** 10.1371/journal.pone.0361574

**Published:** 2026-10-29

**Authors:** Nesibe Şimşekoğlu, İlknur Arslanoğlu, Banu Portakal, Selman Aktaş

**Affiliations:** 1 Department of Fundamentals of Nursing, Hamidiye Faculty of Nursing, University of Health Sciences, İstanbul, Türkiye; 2 Department of Pediatric Endocrinology, Research Hospital, Düzce University Faculty of Medicine, Düzce, Türkiye; 3 Diabetes Polyclinic, Research Hospital, Düzce University Faculty of Medicine, Düzce, Türkiye; 4 Biostatistics Department, Hamidiye Medical Faculty, University of Health Sciences, İstanbul, Türkiye; University of Tabuk, SAUDI ARABIA

## Abstract

**Background:**

Achieving early glycemic control in adolescents with diabetes is associated with improved short- and long-term health outcomes. The aim of this study was to examine the relationship between eating attitudes and glycemic control, as well as the factors associated with eating attitudes and glycemic control, in adolescents with diabetes.

**Methods:**

This descriptive cross-sectional study included 143 adolescents with diabetes, aged 10–19 years, who attended a research hospital between February and April 2024. The data were collected using a Descriptive Information Form, Eating Attitudes Test (EAT) and an information form questioning the values of some metabolic variables.

**Results:**

The mean age of the participants was 14 ± 2.3 years and 49.7% had diabetes between 1–5 years. The mean total score of the EAT scale was 16.62 ± 8.30. The mean score of the “Dieting” subscale of the EAT scale was 10.39 ± 5.58, the mean score of the “Bulimia food preoccupation” subscale was 2.33 ± 3.03 and the mean score of the “Oral Control” subscale was 3.79 ± 3.08. There was no significant difference between the mean EAT total score and glycemic control.

**Conclusions:**

The mean EAT-26 total score was below the commonly used cutoff score, and no significant association was found between EAT-26 scores and HbA1c. Differences in EAT-26 total and subscale scores were observed according to selected demographic and clinical characteristics, including BMI, treatment modality, and treatment adherence. These findings describe self-reported eating attitudes in the study sample and support the inclusion of eating-related assessment within the holistic care of adolescents with Type 1 DM.

## Introduction

Diabetes mellitus (DM) is a common condition that is associated with increasing mortality and morbidity. Type 1 DM is an autoimmune disease characterised by insulin secretion deficiency associated with the destruction of β cells and associated hyperglycemia [1,2]. In the 2022 report of the International Diabetes Federation (IDF), it was reported that there are approximately 8.4 million individuals diagnosed with Type 1 DM worldwide and 1.5 million (18%) of them are under the age of 20. It has been reported that the incidence of Type 1 DM, which is childhood diabetes, is increasing by an average of 3–4% per year [3,4].

The management of Type 1 DM is a complex process that aims to prevent the emergence of acute and chronic complications while maintaining glycemic control [5,6]. Ensuring optimal glycemic control in this process is important for improving short- and long-term health outcomes [7,8]. HbA1c value is an important marker for the evaluation of glycemic control level of individuals [1]. The International Society for Pediatric and Adolescent Diabetes (ISPAD) recommends an HbA1c value below 7.5% (58 mmol/L) as a glycemic control target [4,9,10]. The earlier the HbA1c level is controlled, the greater the favourable effect [9]. As a matter of fact, the Epidemiology of Diabetes Interventions and Complications (EDIC) study, by drawing attention to the concept of metabolic memory, stated that high blood glucose levels in the early years of Type 1 DM will be effective in the development of long-term complications and emphasised the importance of early insulin treatment in preventing these complications. Early and intensive control of diabetes provides protection against macrovascular and microvascular complications and supports the expected growth and development in adolescence. It will also contribute to the reduction of the economic and care burden of the health system and caregivers [4,7].

Despite promising therapeutic advances, such as improved diabetes technology and new adjuvant medications, and improved quality of health care delivery, only 21% of adolescents achieve target glycemic control [4,8,9]. Individuals with Type 1 DM have to deal with many problems in their daily lives to maintain glycemic control. Achieving glycemic control requires adherence to a strict regime including regular attendance at scheduled appointments, carbohydrate calculations, multiple insulin administration, frequent blood glucose measurements, continuous diet planning and exercise programmes [11,12]. However, these demanding self-management tasks may be associated with reduced independence, lower treatment adherence, and greater challenges in diabetes management. These challenges may become even more pronounced during adolescence, a transitional period between childhood and adulthood [5,12,13]. Adolescence, defined by WHO as the period between the ages of 10–19, is a life stage characterized by significant changes in biological, psychological and social dimensions. Factors such as limited problem-solving skills, adolescent rebellion, peer pressure, the transition of diabetes management responsibility from parents to adolescents, and dietary habits may be associated with poorer adherence to diabetes management among adolescents [8,14]. Attitudes and habits related to nutrition established during this period are considered important determinants of adolescent health in both the short and long term [15]. Due to the intense emotional fluctuations seen in adolescence and the physiological burden of Type 1 DM, this period is considered a critical period for the development of eating disorders [16].

A significant cause of difficulty in glycemic control during childhood and adolescence is eating disorders (ED). ‘Eating disorders (ED)’ is a catch-all term for pathological behaviors related to eating. Eating disorders are categorised as Eating Disorders Not Otherwise Specified (EDNOS), which includes specific eating disorders such as Anorexia Nervosa (AN) and Bulimia Nervosa (BN) and all other types of eating disorders [1,17]. Eating disorders in diabetic individuals were first observed in the 1970s. Eating disorders are reported in approximately 20% of women with DM and are approximately twice as common among adolescent girls with Type 1 DM than among their peers without diabetes [18–20]. High BMI in adolescence, body image concerns, presence of conflict in the family, deterioration in mother-daughter relationship, low self-esteem, longer duration of diabetes and poor metabolic control, avoidant and negative coping methods related to the disease increase the risk of eating disorders in diabetic individuals. In addition, the fact that they have to pay attention to meal times, the content of the foods consumed and the amount of energy and carbohydrate in a portion for the regulation of insulin treatments during the diabetes treatment process increases the risk of developing eating disorders in adolescents [6,20]. In the Transdiagnostic Care Model developed by Fairburn (2003), it was reported that characteristics such as low self-esteem and perfectionism trigger eating disorders in Type 1 diabetic individuals [12,21]. Since symptoms of disordered eating including behaviors such as overeating, binge eating, use of laxatives or diuretics, excessive exercise and self-induced vomiting in individuals with Type 1 DM may develop into significant eating disorders over time, it is important to identify these concerns early period and to prepare treatment and care protocols [1,20,22]. Diabulimia, which is described as “The World’s Most Dangerous Eating Disorder”, is characterised by deliberate restriction of insulin that causes weight gain in diabetic individuals [1,23].

Although previous studies have investigated the relationship between disordered eating behaviors and glycemic outcomes in adolescents with Type 1 DM, the available evidence remains limited and inconsistent due to differences in study populations, assessment methods, and sociocultural contexts. Furthermore, most existing studies have primarily focused on clinically diagnosed eating disorders or diabetes-specific eating problems, whereas evidence regarding general eating attitudes and their relationship with glycemic control is still limited, particularly among adolescents from different cultural settings. Therefore, further evidence is needed to clarify this relationship and to better understand the potential role of eating attitudes in diabetes management among adolescents with Type 1 DM [16,24].

By examining the association between eating attitudes and glycemic control in adolescents with Type 1 DM, this study aims to address an important gap in the literature and provide evidence from a Turkish population, where data on this topic remain scarce. The findings are expected to contribute to a better understanding of behavioral factors associated with glycemic control and to inform future research and the development of targeted interventions for improving diabetes management in adolescents. Accordingly, the aim of this study was to examine the relationship between eating attitudes and glycemic control among adolescents with Type 1 DM.

### Research questions

1. Is there a relationship between disordered eating attitudes and glycemic control variables in adolescents with diabetes?
2. Are disordered eating attitudes in adolescents with diabetes associated with demographic and disease-related variables?

## Methods

### Study design

This study was designed as a descriptive cross-sectional study. The STROBE checklist for cross-sectional studies was used in reporting this study.

### Place and time of the study

The study was conducted in the Pediatric Endocrinology Outpatient Clinic of a research hospital between 10/02/2024–26/04/2024.

### Sample

The population of the study consisted of adolescents with diabetes who applied to the Pediatric Endocrinology Outpatient Clinic of a research hospital. Adequate sample size for the study was calculated with the ‘*pwrss*’ package of *RStudio* (*version 9.3, Armonk, NY:IBM Corp*) programme. In the literature review [25], based on the percentage measurement values of the methods to be studied; in the pearson correlation test (double tail); The sample size was calculated as 121 when the error level (*α*)=0.05, the power of the test (*1-β*)=0.80 and *r*1 (minimum correlation coefficient)=0.35 were taken. The study was completed with 143 adolescents with diabetes who agreed to be included in the study. All participants who met the inclusion criteria and agreed to participate in the study were included using a sequential sampling method. Of the 168 participants invited to the study, 143 participated, resulting in a response rate of 85%. There were no missing data in the dataset that would affect the analyses. Forms with missing or incorrectly filled-in information were excluded from the analysis. Due to the cross-sectional study design, there were no cases of loss to follow-up or participants who withdrew from the study during the data collection process.

**Inclusion criteria:**

- Adolescents aged 10–19 years,
- 18 and 19 years old and approved the informed consent form,
- Under 18 years old and approved the informed consent form by their parents,
- Diagnosed with Type 1 DM at least 6 months ago,
- Without visual and hearing impairment,
- Literate,
- Examined in the Pediatric Endocrinology Outpatient Clinic of a research hospital between 10/02/2024-26/04/2024 were included in the study.

### Data collection

Descriptive Information Form, Eating Attitudes Test and information form questioning the values of some metabolic variables were used as data collection tools. The data were collected by the researchers by applying a face-to-face questionnaire to the patients in approximately 5–6 minutes.

### Descriptive information form

The “Descriptive Information Form,” consisting of a total of 28 questions, aims to examine the demographic characteristics of the participants with the first 9 questions, and the following 19 questions with disease-related characteristics.

### Eating attitudes test-26 (EAT-26)

The Eating Attitudes Test-40 (EAT-40) was developed by Garner and Garfinkel (1979) to determine anorectic/bulimic attitudes and behaviors. EAT-40 was later revised by Garner et al. (1982) and a short form with 26 questions was developed. In a pilot study conducted by Devran (2014) using the translated form of EAT-26, Cronbach’s α coefficient was 0.70 and the intraclass correlation coefficient was 0.95. Eating attitudes test-26 consists of 26 items. The items of the scale were prepared in the form of a six-point Likert scale. The items consist of ‘always’, ‘usually’, ‘frequently’, ‘sometimes’, “rarely” and ‘never’ options. Questions 1–25 are evaluated as always = 3; usually = 2; frequently = 1; other options = 0 points. Question 26 is scored in the opposite direction (never = 3, rarely = 2, sometimes = 1, other options = 0). A total score is obtained by scoring all questions. The total score ranges from 0 to 78 and ≥ 20 points is considered as cutoff value. A total score of 20 or above is commonly used as a positive screening threshold indicating the need for further assessment; it does not establish a clinical diagnosis [25].

The EAT-26 consists of three subscales: dieting behavior (1.,6.,7.,10.,11.,12.,14.,16.,17.,22.,23.,24.,26. items), Bulimia and food preoccupation (3.,4.,9.,18.,21.,25.items) and oral control (2.,5.,8.,13.,15.,19.,20. items), allowing more information to be obtained from a single scale. Dieting behavior is highly correlated with an impaired body image. Bulimic behavior is closely related to body weight and provides information about the tendency to bulimic behavior and body perception. Oral control behavior reflects a tendency for self-control. High scores in this domain are associated with the absence of bulimia, and low body weight. Among the subscales of the scale, dieting score is calculated by summing the scores of 13 questions, bulimic behavior score is calculated by summing the scores of 6 questions and oral control behavior score is calculated by summing the scores of 7 questions. The cutoff values for dieting, Bulimia and food preoccupation and oral control subscales are 18, 4 and 5 points, respectively [25].

As a result of the reliability analysis regarding the internal consistency of the Eating Attitudes Test (EAT) scale, the total Cronbach’s α coefficient of the scale was calculated as 0.688. When evaluated according to the subscales, the Cronbach’s α coefficient of the ‘Diet’ subscale was 0.662, the “Bulimia food preoccupation” subscale was 0.534 and the ‘Oral Control’ subscale was 0.549. These results show that especially the total scale and the ‘Diet’ subscale have an acceptable level of internal consistency.

### Glycemic control variables information form

This form was used to assess metabolic control variables, including HbA1c and BMI values. HbA1c values were obtained from laboratory records during the same clinical visit as the questionnaire administration and were not based on participant self-report. Glycemic control was classified according to HbA1c levels as good (<7.5%) or poor (≥7.5%), in accordance with the International Society for Pediatric and Adolescent Diabetes (ISPAD) guidelines.

### Analysis of data

Statistical analyses in this study were performed using SPSS 25.0 (Released 2017. IBM SPSS Statistics for Windows, Version 25.0. Armonk, NY: IBM Corp) and visualization was performed using RStudio version 2024.12.1 + 563 ‘Kousa Dogwood’ (Windows 10 based system) and Quarto 1.5.57 platform.

The distribution characteristics of the data were evaluated with descriptive statistics, and the normality assumption was checked. Since the variables did not show normal distribution, nonparametric methods were preferred in the analyses. The differences of EAT total score and subscale scores (Diet, Bulimia food preoccupation and Oral Control) according to demographic and disease-related variables were analyzed by Kruskal-Wallis H test. In cases where a significant difference was found as a result of the Kruskal-Wallis test, within-group pairwise comparisons were made with the Dunn-Bonferroni post hoc test. In addition, Spearman’s rank-order correlation difference correlation analysis was used to examine the relationships between EAT and its subscale scores and continuous variables (age, HbA1c level and BMI). To evaluate whether eating attitudes were independently associated with glycaemic control, a multivariable linear regression analysis was performed with HbA1c as the dependent variable and EAT-26 total score, age, gender, BMI z-score, diabetes duration, dietary adherence, physical activity, and meal skipping as independent variables. Statistical significance level was accepted as p < 0.05.

### Ethics statement

The ethics committee of the University of Health Sciences approved the current study (Decision No: 23–705), which was conducted in accordance with the ethical code of the Declaration of Helsinki. In the light of the principle of voluntariness, written informed consent was obtained from all participants aged 18 years or older. For participants under 18 years of age, written informed consent was obtained from their parents or legal guardians, and written assent was obtained from the adolescents prior to their participation in the study.

## Results

The findings related to the socio-demographic and disease characteristics of the individuals included in the study are given in Table 1. Accordingly, it was found that the mean age of the participants included in the study was 14 ± 2.3, 81(56.6%) were female, 5(3.5%) were married, 71(49.7%) were primary school graduates and 92(64.3%) had a family income equivalent to their expenses.

**Table 1:** Findings regarding the socio-demographic and disease characteristics of individuals (n = 143).

Variable		n(%)
**Gender**	Female	81(56.6)
	Male	62(43.4)
**Marital status**	Married	5(3.5)
	Single	138(96.5)
BMI-for-age z-score*	Severe thinness (<−3 SD)	1(0.7)
	Thinness (−3 to <−2 SD)	1(0.7)
	Normal (−2 to +1 SD)	78(54.5)
	Overweight (>+1 to +2 SD)	42(29.4)
	Obesity (>+2 SD)	21(14.7)
**Educational background**	Literate	14(9.8)
	Primary education	71(49.7)
	High school	58(40.6)
**Family income status**	Income is less than expenses	33(23.1)
	Income exceeds expenses	18(12.6)
	Income equals expenses	92(64.3)
**Chronic disease other than diabetes**	No	129(90.2)
	Yes	14(9.8)
**Diabetes diagnosis time**	<1 years	18 (12.6)
	1-5 years	71(49.7)
	6-10 years	39(27.3)
	11-20 years	15(10.5)
**Diabetes control frequency**	Once or twice a month	16(11.2)
	Three times a month or more	127(88.8)
**Family history of diabetes**	No	76(53.1)
	Yes	67(46.9)
**Compliance with treatment**	Good	47(32.9)
	Moderate	81(56.6)
	Poor	15(10.5)
**Compliance with diet**	Yes	24(16.8)
	No	34(23.8)
	Sometimes	85(59.4)
**Regular exercise**	Yes	34(23.8)
	No	48(33.6)
	Sometimes	61(42.7)
**Number of daily main meals (breakfast, lunch, dinner)**	Two times	38(26.5)
	Three times or more	105(73.5)
**Number of snacks per day**	Once	23(16.1)
	Two times	43(30.1)
	Three times	20(14.0)
	More than three	46(32.2)
	Does not consume	11(7.7)
**Eating speed**	Slow	11(7.7)
	Medium	87(60.8)
	Fast/Very fast	45(31.5)
**Meal skipping**	Yes	39(27.3)
	No	44(30.8)
	Sometimes	60(42)
**Habit of waking up and eating at night**	Yes	9(6.3)
	No	116(81.1)
	Sometimes	18(12.6)
**Consuming food outside the home**	Every day	20(14)
	Every other day	12(8.4)
	1-2 times a week	29(20.3)
	Once every 15 days	28(19.6)
	Once a month	43(30.1)
	Never	11(7.7)
**Fast food consumption**	Every day/ Every other day	11(7.7)
	1-2 times a week	42(29.4)
	Once every 15 days	26(18.2)
	Once a month	46(32.2)
	Never	18(12.6)
**Consumption of soft drinks**	Every day/ Every other day	22(15.4)
	1-2 times a week	33(23.1)
	Once every 15 days	38(26.6)
	Once a month	33(23.1)
	Never	17(11.9)
**Age**	**Mean+Sd**	**Med(Min-Max)**
	14 + 2.3	14(10-19)

* BMI*-for-age categories were classified according to the 2007* World Health Organization (WHO*) Growth*

*Reference for children and adolescents aged 5–19 years.*

When the data of the participants regarding diabetes disease were examined, it was found that 71 (49.7%) of them had a diagnosis of DM between 1–5 years, 67 (46.9%) had a family history of diabetes, and 14 (9.8%) had other chronic diseases other than diabetes. 81(56.8%) of the participants rated their compliance with treatment as “moderate”, 34(23.8%) did not comply with the diet and 48(33.6%) did not exercise regularly.

When the nutritional characteristics of the participants were analyzed, 105(73.5) of them ate three main meals a day, 46 (32.2%) consumed more than three snacks a day, 87 (%60,8) had a moderate eating rate, 39 (27.3%) skipped meals, 46 (32.2%) consumed fast food once a month and only 17 (11.9%) did not consume acidic drinks. In addition, the BMI z scores of the participants were found between −3.02 and 3.19.

The mean total score of the participants EAT scale and subdimensions were shown in Table 2. The mean score of the “Dieting” subscale, the mean score of the “Bulimia food preoccupation” subscale and the mean score of the “Oral Control” subscale were found to be 10.39 ± 5.58, 2.33 ± 3.03 and 3.79 ± 3.08, respectively. The mean total EAT score was found to be 16.62 ± 8.30. And 28.7% (41) of the participants had a mean EAT score exceeding the threshold value of 20.

**Table 2:** EAT scale and subscale averages (n = 143).

	Mean±Sd	Med. (Min-Max)
Dieting	10.39 ± 5.58	1-27
Bulimia food preoccupation	2.33 ± 3.03	0-20
Oral Control	3.79 ± 3.08	0-15
EAT	16.62 ± 8.30	3-41

According to the Spearman correlation analysis results presented in Fig 1, a significant and strong positive correlation was found between the EAT total score and the Diet subscale (ρ = 0.82, p < 0.05). In addition, a significant, moderate positive correlation was observed between EAT total score and Oral Control (ρ = 0.68, p < 0.05) and Bulimia food preoccupation (ρ = 0.52, p < 0.05) subscales. A significant but weak positive relationship was found between Oral Control and Diet (ρ = 0.39, p < 0.05) and Oral Control and Bulimia food preoccupation (ρ = 0.21, p < 0.05) subscales. On the other hand, no statistically significant correlation was found between EAT and its subscales and HbA1C, age and BMI (p > 0.05). A significant and moderate positive correlation was found between age and BMI (ρ = 0.49, p < 0.05).

**Fig 1:**
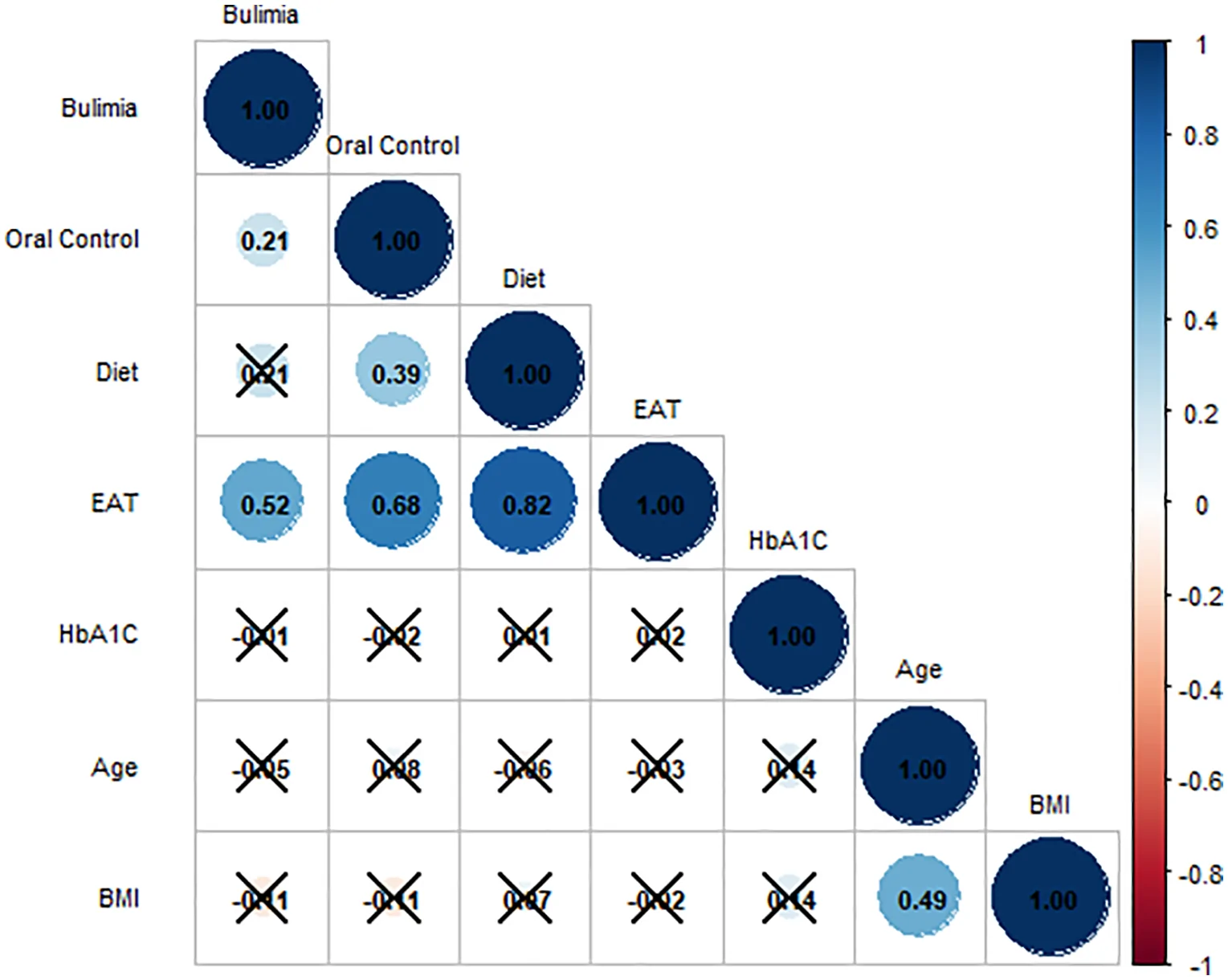
Spearman Correlations Between EAT Scores and HbA1C, Age, and BMI.

The distributions of the total and subscale mean scores of the participants according to various demographic and disease characteristics were evaluated both with statistical test results Table 3, Fig 2 and Fig 3. Accordingly, a significant difference was found in the ‘Diet’ subscale scores according to the gender variable (p = 0.002). ‘Diet’ scores of female participants were higher than male participants. A significant difference was observed in the ‘Bulimia food preoccupation’ subscale according to smoking status (p = 0.007). ‘Bulimia food preoccupation’ scores of smokers were higher than non-smokers and individuals who had smoked in the past. Significant differences were found between the level of compliance with diabetes treatment and ‘Diet’ (p = 0.019), ‘Oral Control’ (p = 0.004) and total EAT scores (p = 0.036). Especially in the ‘Oral Control’ subscale, the scores of individuals with good treatment compliance were higher than those with moderate compliance. Exercise status showed a significant difference in the ‘Bulimia food preoccupation’ subscale (p = 0.023). ‘Bulimia food preoccupation’ scores of individuals who exercised regularly were higher than those of individuals who did not exercise. A significant difference was found between compliance with the diabetes diet and ‘Oral Control’ (p = 0.005) and total EAT score (p = 0.004). The scores in these two scales were found to be higher in individuals who followed the diet compared to individuals who did not follow the diet. A significant difference was found in total EAT scores according to meal skipping status (p = 0.036). The total EAT scores of individuals who reported skipping meals were higher than those of individuals who reported skipping meals only occasionally. A significant difference was found between the ‘Bulimia food preoccupation’ subscale scores according to body mass index categories (p = 0.047), but no significant difference was found between the groups according to post hoc analysis results. Moreover, a significant difference was found between compliance with the eating speed (p = o.o44), soft drink consumption (p = 0.001) and ‘Bulimia food preoccupation’ score. A significant association was also found regarding soft drink consumption (p = 0.034) within the ‘Diet’ subscale (Fig 3).

**Table 3:** Evaluation of EAT and its subscales in terms of demographic and disease characteristics (n = 143).

	Diet	Bulimia food preoccupation	Oral Control	EAT
***Gender***				
Male	8(1-24)	1.5(0-11)	3(0-15)	15.5(3-41)
Female	10(3-27)	0(0-20)	3(0-6)	14(7-37)
***p***	0.002*	0.477	0.926	0.093
***Effect size***	-0.302	0.068	-0.009	-0.164
***95% CI***	-0.477-0.124	-0.112–0.257	-0.198–0.186	-0.348–0.024
***Smoking Status***				
Non-smoker	8.5(1-27)	1(0-20)a	3(0-15)	14(3-41)
Former smoker	12(12-12)	0(0-0)a	6(6-6)	18(18-18)
Smoker	13(11-14)	7(3-7)b	7(2-10)	26(17-28)
***p***	0.154	0.007*	0.084	0.051
***Effect size***	0.026	0.069	0.035	0.042
***95% CI***	0.011–0.047	0.045–0.100	0.015–0.088	0.015–0.076
***Time to diagnosis of Diabetes***				
<1 years	7(3-23)	3(0-20)	3.5(0-11)	16(4-41)
1-5 years	9(3-27)	1(0-11)	3(0-15)	17(4-37)
6-10 years	9(1-23)	2(0-8)	3(0-12)	13(4-31)
11-20 years	8(3-24)	1(0-3)	2(0-6)	11(3-26)
***p***	0.076	0.058	0.309	0.076
***Effect size***	0.048	0.052	0.025	0.048
***95% CI***	0.010–0.156	0.014–0.153	0.005–0.114	0.014–0.157
***Adherence to Diabetes Treatment***				
Good	10(3-23)a	1(0-10)	5(0-15)a	18(4-41)a
Moderate	8(1-27)a	2(0-20)	3(0-12)b	13(3-37)a
Poor	13(5-19)a	3(0-11)	3(0-10)ab	17(5-33)a
***p***	0.019*	0.314	0.004*	0.036*
***Effect size***	0.056	0.016	0.076	0.047
***95% CI***	0.011–0.157	0.001–0.103	0.016–0.188	0.008–0.150
***Regular Exercise***				
Yes	10(3-23)	2(0-10)b	3(0-12)	16.5(4-41)
No	8.5(3-21)	0.5(0-11)a	3(0-10)	12.5(3-33)
Sometimes	9(1-27)	2(0-20)ab	4(0-15)	16(5-37)
**p**	0.907	0.023*	0.476	0.307
***Effect size***	0.001	0.053	0.010	0.017
***95% CI***	0.000–0.054	0.009–0.150	0.001–0.084	0.001–0.093
***Compliance with diet***				
Yes	9(6-23)	2(0-10)	5(0-15)b	21(6-41)b
No	8(3-17)	1(0-5)	2(0-10)a	13(3-27)a
Sometimes	9(1-27)	1(0-20)	3(0-12)a	16(4-37)ab
**p**	0.071	0.531	0.005*	0.004*
***Effect size***	0.037	0.009	0.074	0.077
***95% CI***	0.006–0.119	0.001–0.070	0.016–0.184	0.019–0.185
***Skipping meals***				
Yes	11(3-23)	2(0-11)	4(0-15)	17(3-36)b
No	8.5(3-23)	1(0-10)	4(0-11)	16(4-41)ab
Sometimes	8(1-27)	1(0-20)	3(0-12)	13(4-37)a
***p***	0.057	0.228	0.185	0.036*
***Effect size***	0.040	0.021	0.024	0.047
***95% CI***	0.005–0.130	0.002–0.107	0.002–0.104	0.007–0.140
***BMI***				
Underweight	8(1-24)	1.5(0-11)a	3(0-15)	15.5(3-41)
Normal	10(3-27)	0(0-20)a	3(0-6)	14(7-37)
Overweight	11.5(5-21)	2.5(0-6)a	3(0-10)	16.5(5-26)
**p**	0.604	0.047*	0.621	0.841
***Effect size***	0.007	0.043	0.007	0.002
***95% CI***	0.001–0.061	0.008–0.125	0.001–0.059	0.000–0.045

Note: Data are presented as median(minimum-maximum). *p < 0.05. Effect size is Cliff’s delta for two-group comparisons and epsilon squared (epsilon²) for comparisons with more than two groups. CI: confidence interval. Groups sharing the same superscript letter are not significantly different from each other; different letters indicate significant differences according to Bonferroni-adjusted pairwise comparisons.

**Fig 2:**
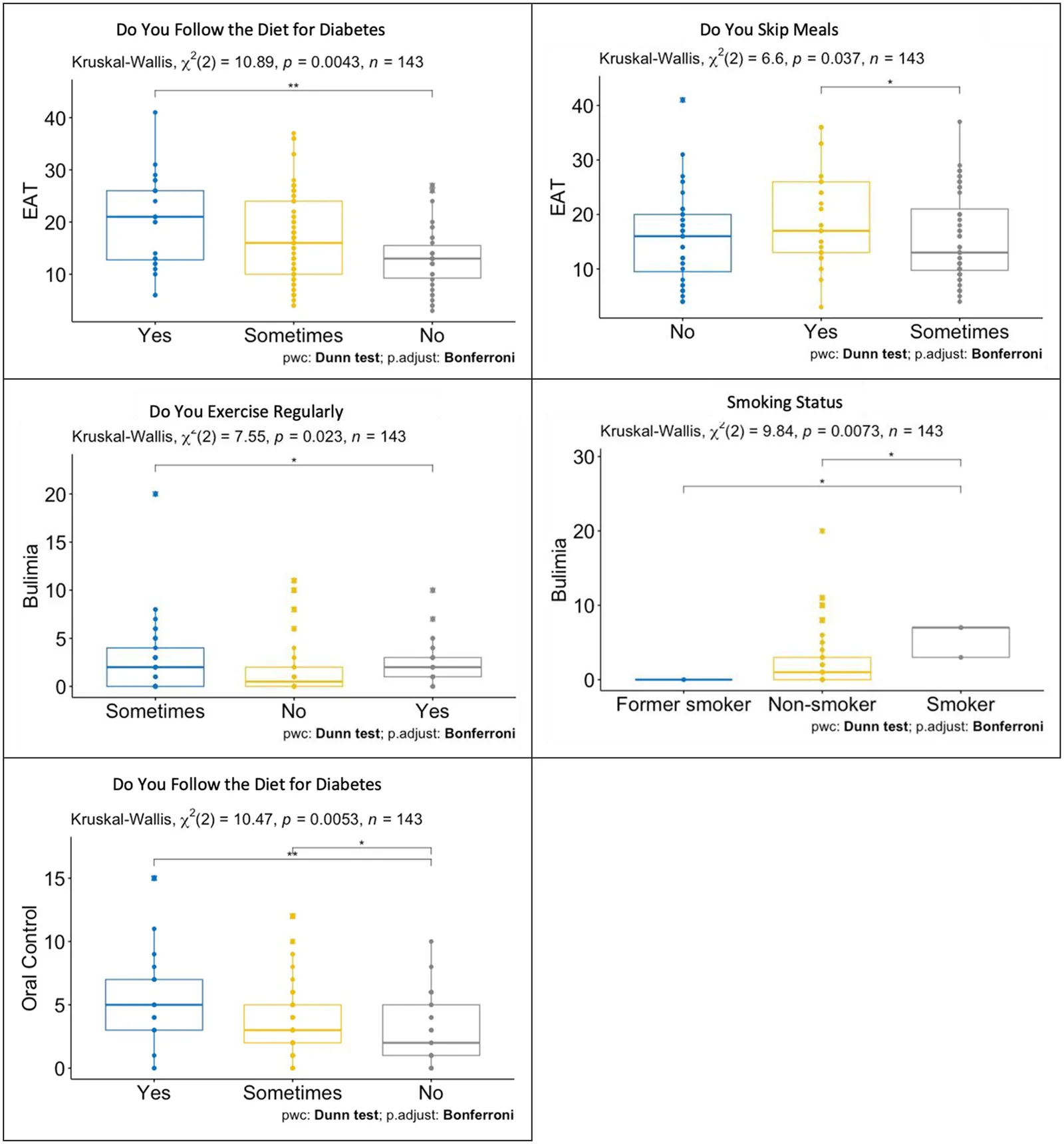
Comparison of participants’ EAT and subscale scores according to demographic and disease characteristics.

**Fig 3:**
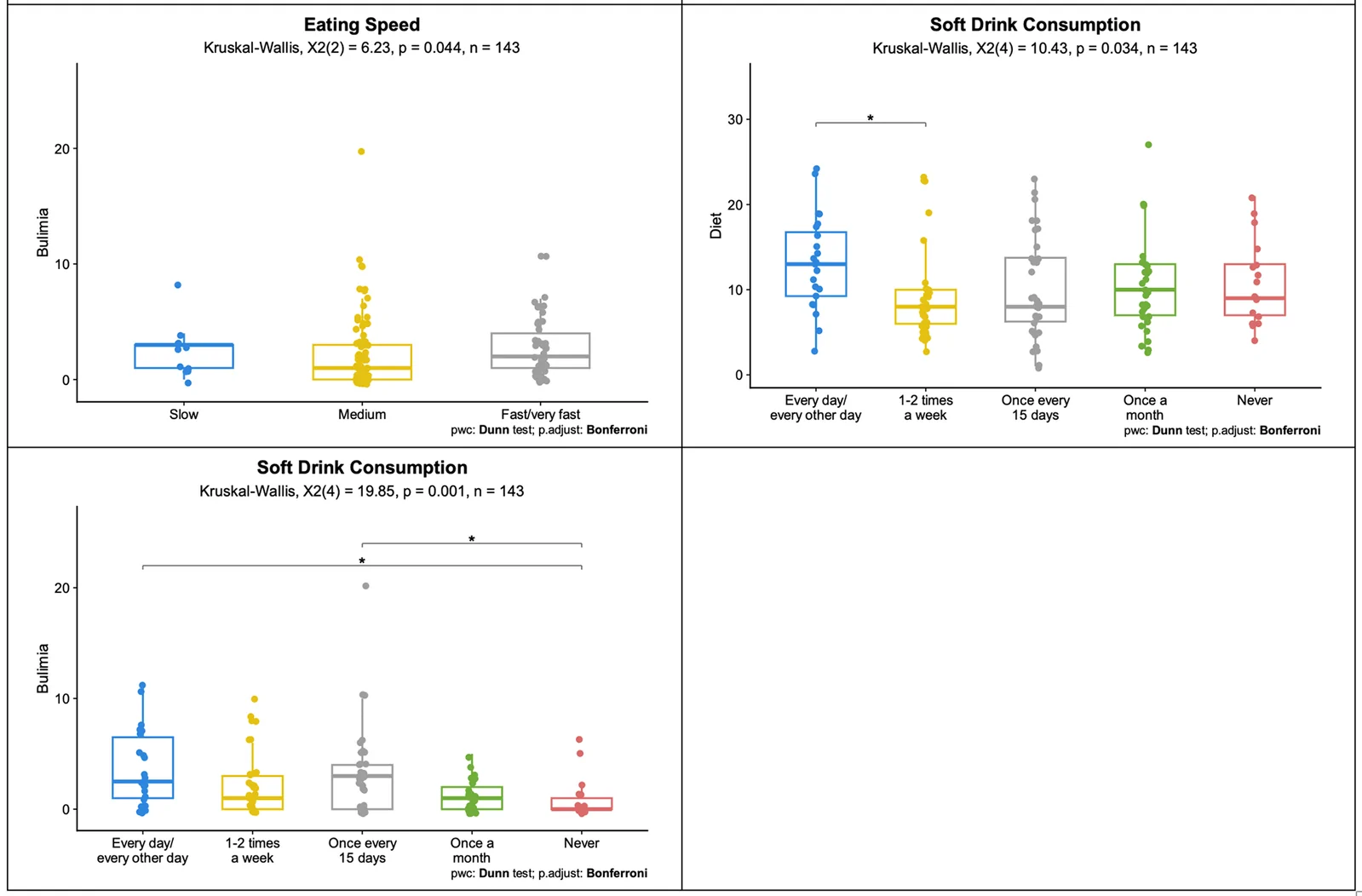
Comparison of participants’ EAT and subscale scores according to demographic characteristics.

A forced-entry multivariable linear regression analysis was performed with HbA1c as the dependent variable (Table 4). The model included EAT-26 total score, age, gender, BMI category, diabetes duration, dietary adherence, physical activity, and meal skipping. Categorical variables were entered using dummy coding. The overall model was not statistically significant (R² = 0.084, adjusted R²= −0.017, F = 0.834, p = 0.631). After adjustment for the included demographic and clinical variables, the EAT-26 total score was not independently associated with HbA1c (B = 0.018, standardized β = 0.085, 95% CI: −0.022 to 0.058, p = 0.372).

**Table 4:** Multivariable linear regression analysis of factors associated with HbA1c (n = 143).

Variable	B	SE	β	95% CI	p
EAT-26 total score	0.018	0.020	0.085	−0.022 to 0.058	0.372
Age (years)	0.106	0.062	0.164	−0.018 to 0.229	0.092
BMI z-score	0.045	0.256	0.017	−0.462 to 0.551	0.862
Gender	0.103	0.315	0.031	−0.520 to 0.726	0.745
Diabetes duration	0.045	0.195	0.022	−0.340 to 0.431	0.816
Dietary adherence	−0.249	0.200	−0.120	−0.644 to 0.145	0.214
Regular physical activity	−0.276	0.198	−0.134	−0.667 to 0.116	0.166
Meal skipping	0.317	0.198	0.153	−0.075 to 0.708	0.112

*B = unstandardized regression coefficient; SE = standard error; β = standardized regression; coefficient; CI = confidence interval; R^2^ = 0.084; adjusted R^2^ = −0.017.*

## Discussion

This study examined the relationship between eating attitudes and glycemic control variables in adolescents with Type 1 DM (Table 2). The mean EAT-26 total score was 16.62 ± 8.30, which was below the commonly used cutoff score of 20. At the group level, this finding may indicate that the eating-related concerns assessed by the EAT-26 were not prominent in this sample [14,26]. Nevertheless, individual scores may vary, and the mean score provides only an overall description of the sample. Previous studies using the same instrument have reported varying EAT-26 scores across different populations. Higher EAT-26 scores were reported among individuals with multiple sclerosis and hypertension [27,28], whereas lower scores were reported among medical and nursing students and adolescents [29–32]. These differences may be associated with variations in demographic and clinical characteristics, sociocultural factors, and study methods, highlighting the multifactorial nature of eating attitudes.

The mean scores obtained from the EAT-26 subscales provided descriptive information about different dimensions of the participants’ self-reported eating attitudes. The Bulimia and Food Preoccupation and Oral Control subscale scores reflected the participants’ responses to the eating-related attitudes assessed within these dimensions. These scores represent scale-based descriptions at the group level rather than clinical evaluations of body image, bulimia, obesity, or self-control. Studies using the EAT-26 generally report the total mean score, whereas subscale mean scores are reported less frequently [31,33]. Nevertheless, some studies have reported subscale score patterns comparable to those observed in the present study [34–36]. In a study examining the role of procrastination in the relationship between emotional abuse and eating behavior among adolescents, the EAT subscale scores showed a pattern similar to that observed in the present sample [29].

The relationships between the EAT-26 total and subscale mean scores indicated that the different dimensions of eating attitudes were interrelated. In particular, the Dieting subscale showed a stronger association with the total score, suggesting that dieting-related attitudes were more prominent in the overall EAT-26 score in this sample [36]. The associations of the total score with the Oral Control and Bulimia and Food Preoccupation subscales also reflected the multidimensional nature of the eating attitudes assessed by the EAT-26. However, the more limited associations reported among the subscales in some studies suggest that these relationships may vary according to sample characteristics [37].

The Oral Control subscale showed limited associations with the Dieting and Bulimia and Food Preoccupation subscales. This pattern suggests that perceived control over eating may represent a relatively distinct dimension of eating attitudes. Similar findings in previous studies indicate that the dimensions assessed by the EAT-26 may be related to one another to varying degrees [26,38].

The EAT-26 total and subscale mean scores were evaluated according to the participants’ demographic and clinical characteristics (Table 3 and Fig 2). No significant association was found between EAT-26 scores and HbA1c, an indicator of glycemic control. Similarly, the multivariable linear regression analysis showed that EAT-26 scores were not independently associated with HbA1c after adjustment for demographic and clinical variables. BMI was positively associated with age, which may reflect changes in body weight and composition during adolescence. Previous studies have reported associations between eating-related measures and triglyceride levels, BMI, diabetes duration, and HbA1c [39]. In contrast to studies reporting an association between eating attitudes and HbA1c, the present study found no significant relationship between these variables. This difference may be related to variations in sample characteristics, age groups, measurement instruments, or study designs.

The findings should also be considered in relation to the distinction between eating attitudes and overall dietary quality. A recent cross-sectional study conducted among children and adolescents with Type 1 DM in Türkiye reported that a more pro-inflammatory dietary profile, assessed using the Dietary Inflammatory Index, was associated with poorer glycemic control and a less favorable cardiometabolic and inflammatory profile [40]. This evidence suggests that broader aspects of dietary quality and dietary adherence may be more closely related to metabolic control than the eating attitudes assessed by the EAT-26. Thus, although EAT-26 scores were not significantly associated with HbA1c in the present study, other dietary characteristics may still contribute to glycemic control.

Female participants had higher Dieting subscale scores than male participants. This finding is consistent with studies reporting higher EAT-26 scores among females [17,41–45]. However, some studies have reported higher scores among males [46,47]. Differences in findings according to gender may be related to sample characteristics, sociocultural factors, and variations in the measurement instruments used.

Significant differences were observed in the Dieting, Oral Control, and total EAT-26 scores according to the level of adherence to diabetes treatment. In particular, participants with good treatment adherence had significantly higher Oral Control scores. This finding may reflect a relationship between treatment adherence and the control-related eating attitudes assessed by the Oral Control subscale. Previous studies examining diabetes self-care have also demonstrated relationships among treatment adherence, clinical outcomes, and self-care behaviors. Lower medication adherence was associated with higher BMI and HbA1c levels in a study of medication adherence and diabetes self-care activities [48]. Another study conducted in Greece reported that the association between treatment non-adherence and poor glycemic control was stronger among individuals receiving insulin therapy than among those using oral hypoglycemic agents [49].

A significant overall difference was observed in the Bulimia and Food Preoccupation subscale scores across BMI categories; however, the post hoc analysis did not identify a significant difference between any specific pair of groups. Thus, the overall difference could not be attributed to a particular BMI category. This finding partially aligns with the study by Troncone et al. (2018), which reported no significant association between BMI and eating-related measures [13]. By contrast, other studies have reported associations of BMI and HbA1c with eating-related measures [16,41,50].

Previous studies have linked eating attitudes with a broad range of demographic and behavioral factors, including gender, age, place of residence, BMI, parental education, childhood obesity, experiences of bullying, dietary habits, sleep problems, and perceptions of weight and body image [32, 42, 46, 50, 51]. Psychological and social factors, including anxiety, depression, childhood trauma, social and emotional loneliness, self-esteem, problematic use of digital technologies, and perfectionism, have also been associated with eating-related attitudes and behaviors [21,29–31,46,52–54]. Findings may also vary according to the characteristics and circumstances of the population examined. For example, Ramadan fasting was not associated with daily energy intake, irregular eating behaviors, or body image dissatisfaction among adolescents [55], whereas professional and amateur dancers with eating disorders were reported to have higher levels of pain and anxiety [56]. Collectively, these findings illustrate the diverse factors associated with eating attitudes and suggest that differences across studies may partly reflect the demographic, behavioral, psychological, and sociocultural characteristics of their samples. Because these factors were not all assessed in the present study, direct comparisons with these findings remain limited.

These findings have implications for fundamental nursing care, particularly in relation to holistic, person-centered, and developmentally appropriate assessment and support. In adolescents with Type 1 DM, the assessment of eating attitudes alongside treatment adherence, glycemic indicators, growth, and psychosocial well-being can provide a more comprehensive understanding of individual care needs. Through systematic observation, therapeutic communication, health education, and ongoing follow-up, nurses can recognize eating-related concerns and support adolescents in developing appropriate self-care behaviors. When additional evaluation is needed, nurses can also facilitate timely collaboration with other members of the healthcare team. Integrating these components into routine nursing care may strengthen individualized care planning and promote continuity of diabetes management during adolescence [16,17,31].

The present study addresses an underexplored area in Türkiye by examining eating attitudes in relation to glycemic control among adolescents with Type 1 DM. Nevertheless, the single-center design and limited representativeness of the sample may restrict the applicability of the findings to broader adolescent populations. The use of self-report questionnaires may have introduced response and information biases, while the cross-sectional design does not allow causal relationships to be established. The relatively low internal consistency coefficients of the Bulimia and Food Preoccupation and Oral Control subscales may also have affected the stability of the corresponding subscale findings. As the EAT-26 is a screening instrument, combining it with clinical interviews and other assessment methods in larger, multicenter studies may provide a more comprehensive understanding of eating attitudes among adolescents with Type 1 DM.

## Conclusions

No significant association was found between EAT-26 scores and HbA1c among adolescents with Type 1 DM in this cross-sectional sample. The relationships observed among the EAT-26 total and subscale scores reflect the multidimensional nature of self-reported eating attitudes. Assessing eating-related concerns as part of routine clinical care may help identify adolescents who could benefit from a more comprehensive evaluation and individualized support. A holistic assessment that considers biological, psychosocial, behavioral, and sociocultural factors may contribute to a better understanding of eating attitudes among adolescents with Type 1 DM. Integrating the assessment of eating attitudes into routine nursing care may support individualized care planning, appropriate self-care behaviors, and continuity of diabetes management during adolescence.

## Abbreviations

EAT: Eating Attitudes Test
BMI: Body mass index
Type 1 DM: Type 1 Diabetes
IDF: International Diabetes Federation
HbA1c: Glycated hemoglobin
β cells: Beta cells
ISPAD: International Society for Pediatric and Adolescent Diabetes
WHO: World Health Organization
ED: Eating disorders
EDNOS: Eating Disorders Not Otherwise Specified
AN: Anorexia Nervosa
BN: Bulimia Nervosa
DKA: Diabetic ketoacidosis
EAT-40: The Eating Attitudes Test-40
EAT-26: Eating Attitudes Test-26
MS: Multiple sclerosis
DBM: Doubly burdened malnutrition
MDMA: Methylenedioxymethamphetamine
